# Ethical perspectives of the involvement of vulnerable populations in health research: Elin Lampa

**DOI:** 10.1093/eurpub/ckac131.222

**Published:** 2022-10-25

**Authors:** E Lampa, G Warner, A Sarkadi, A Perez Aronsson, M Thell, U Kihlbom

**Affiliations:** 1 Child Health and Parenting, Uppsala University, Uppsala, Sweden; 2 Centre for Research Ethics and Bioethics, Uppsala University, Uppsala, Sweden

## Abstract

**Background:**

Public involvement in research has potential to transform public health research processes and outcomes, as well as contribute to sustainable collaborations between academia and the civil society. However, this all relies on public involvement being conducted in an ethical and inclusive way, especially when involving representatives from vulnerable populations.

**Methods:**

In this empirically informed theoretical reflection, ethical perspectives on involvement of vulnerable populations in health research were explored using data collected within a public involvement evaluation project. By analysing observational and longitudinal qualitative data from research projects involving public representatives from vulnerable groups, ethical aspects were identified.

**Results:**

Responsibility and decision-making appeared as important ethical aspects, where laws and regulations conflicted with involvement ideals. Similarly, reimbursement and recognition for public contributors became an ethical issue when facing legislation and bureaucracy, especially when involving children or refugees. Another ethical aspect concerned researchers’ concerns in balancing involvement and protection of vulnerable groups, especially when involving contributors living under unstable circumstances. Finally, effectively communicating around research and involvement in an accessible way, for contributors to be involved but not burdened, was a challenge for researchers.

**Conclusions:**

Public involvement of vulnerable populations led to ethical challenges related to conflicting ideals and practical realities, including balancing involvement and protection of contributors. This highlighted a need for ethical guidance to support ethical decision-making and practice. The findings are used to guide the development of an ethical framework for decision-making in public involvement.

**Key messages:**

• Researchers involving public contributors from vulnerable groups face ethical challenges which causes barriers to involvement.

• There is need for guidance on ethical decision-making for researchers involving representatives from vulnerable groups in research.

